# Characterization of Fibrin and Collagen Gels for Engineering Wound Healing Models

**DOI:** 10.3390/ma8041636

**Published:** 2015-04-10

**Authors:** Oihana Moreno-Arotzena, Johann G. Meier, Cristina del Amo, José Manuel García-Aznar

**Affiliations:** 1 Multiscale in Mechanical and Biological Engineering (M2BE), Department of Mechanical Engineering, Aragon Institute of Engineering Research (I3A), Universidad de Zaragoza, Mariano Esquillor Street, 50018 Zaragoza, Spain; E-Mails: oihana.moreno@unizar.es (O.M.-A.); cdelamo@unizar.es (C.A.); 2 ITAINNOVA Instituto Tecnológico de Aragón, 7-8 María de Luna Street, 50018 Zaragoza, Spain; E-Mail: jmeier@itainnova.es

**Keywords:** wound healing, 3D, *in vitro*, hydrogel, scaffold, biomechanical, biophysical, microstructure, permeability, rheology

## Abstract

Hydrogels are used for 3D *in vitro* assays and tissue engineering and regeneration purposes. For a thorough interpretation of this technology, an integral biomechanical characterization of the materials is required. In this work, we characterize the mechanical and functional behavior of two specific hydrogels that play critical roles in wound healing, collagen and fibrin. A coherent and complementary characterization was performed using a generalized and standard composition of each hydrogel and a combination of techniques. Microstructural analysis was performed by scanning electron microscopy and confocal reflection imaging. Permeability was measured using a microfluidic-based experimental set-up, and mechanical responses were analyzed by rheology. We measured a pore size of 2.84 and 1.69 μm for collagen and fibrin, respectively. Correspondingly, the permeability of the gels was 1.00·10^−12^ and 5.73·10^−13^ m^2^. The shear modulus in the linear viscoelastic regime was 15 Pa for collagen and 300 Pa for fibrin. The gels exhibited strain-hardening behavior at *ca.* 10% and 50% strain for fibrin and collagen, respectively. This consistent biomechanical characterization provides a detailed and robust starting point for different 3D *in vitro* bioapplications, such as collagen and/or fibrin gels. These features may have major implications for 3D cellular behavior by inducing divergent microenvironmental cues.

## 1. Introduction

Wound healing demonstrates the capacity of skin to regenerate in an orchestrated manner. However, pathological healing processes, such as fibrosis, hypertrophic scars or ulcers, can lead to major disabilities or even death and have a high global incidence [1]. As a reference, 3–6 million people in the United States were affected by these disorders in 2010 [2].

The healing process is the result of a complex interaction of many factors that regulate the development of wounds. This complexity has been addressed by means of diverse approaches. *In vivo* [3], *in vitro* [4,5] and *in silico* [6] studies have been performed to elucidate fundamental wound healing mechanisms. *In vitro* assays have been developed to analyze reepithelialization [7,8]. However, a more complex 3D process occurs in full-thickness injury healing [9]. Novel methodologies based on microfluidic techniques are being developed that offer unique features for the rational design of physiologically relevant *in vitro* systems [10], which could be directed to mimic wound healing processes. Hydrogels have been employed to resemble the extracellular matrix (ECM) [11,12]. Although progress has been made, knowledge of the most adequate conditions to recreate the local microenvironment of wound healing remains lacking.

In a reductionist simplification of the environmental complexity of the wound healing scenario, three main actors can be distinguished: cells, environmental signaling and the ECM. Most previous studies have focused on analyzing the cell-environment signaling interaction [13]. The regulatory role of the ECM is considered critical for cellular processes, e.g., wound healing [14,15]. The ECM is a 3D fibrillar network that provides both architectural scaffolding and a heterogeneous signaling distribution in the whole cell vicinity. The biomechanical cues arising from the ECM play a fundamental role in the modulation of cellular behavior and mechanotransduction in wound healing [15]. The contributions of matrix stiffness and microstructure on cell behavior have been widely demonstrated [16,17]. Recent evidence also indicates a relevant role of interstitial flow [18,19,20] and ECM confinement level [21,22,23] on basic cellular processes, e.g., 3D cell migration. Therefore, to develop accurate biomimetic *in vitro* models, it is crucial to select the most adequate material to resemble the ECM *in vivo*.

In wound healing, the primary matrices are the fibrin clot and the granulation tissue, which is mainly formed by newly-deposited collagen [15]. To represent these local microenvironments, biomimetic hydrogels composed of fibrin or collagen I, respectively, are typically used [24,25]. These proteins are very useful because they self-assemble at a proper ionic strength [26]. However, for applications with the objective of recreating wound healing environments, a profound knowledge of the biomechanical and biophysical properties of both hydrogels is required.

In this regard, multiple studies have analyzed the microstructural features and bulk stiffness of similar hydrogels [27,28,29,30]. As elements of the microstructure, fiber arrangement and diameter have been extensively studied by scanning electron microscopy (SEM) [31] and confocal reflection imaging (CRI) [32]. Rheological [29,30,33,34], axial tensile tests [28,35] and other techniques [36,37], as well as assays at the individual fiber level [38,39] have been performed. A limited number of studies have also quantified the hydraulic resistance of gel scaffolds to fluid flow. These studies have focused on improving the nutrient diffusion in scaffolds for tissue engineering applications [40,41] or analyzing 3D cell migration [18,42].

The application of collagen and fibrin hydrogels as scaffolds in tissue engineering and *in vitro* experiments and their biomechanical characterization have increased remarkably [27,29,35]. However, these studies have employed a wide diversity of hydrogel compositions and different measurement methods [43]. Modification of the gel composition, polymerization temperature or pH alters various biophysical properties [44]. These variations hinder the application of hydrogels in the controlled representation of microenvironments for wound healing experiments, as well as the analysis of the impact of these parameters on the cell response.

Due to the infeasibility of addressing all possible combinations, in this work, we chose generalized and defined compositions for the gel scaffolds. The chosen specific collagen and fibrin gels have been widely used as physiologically-relevant matrix representations [33,43,45], in applications such as wound healing. Our main aim was to establish a quantitative evaluation of the functional behavior of both hydrogels under experimental conditions that were as comparable as possible. Therefore, we assessed not only the microstructural and rheological properties of both scaffolds, but also their hydraulic resistance to fluid flow. We obtained coherent and corresponding datasets for each hydrogel composition using four complementary experimental techniques that have not previously been used in combination for these gels. This work reports the complete characterization of relevant parameters for biomimetic matrices for 3D *in vitro* assays and primarily focuses on mimicking wound regeneration using widely-used collagen and a fibrin hydrogel compositions. The presented methodology could also be suitable for the study of other scaffolds and their variations.

## 2. Experimental Section

### 2.1. Preparation of Fibrin and Collagen Gels

#### 2.1.1. Fibrin Gels

Plasminogen-, fibronectin- and factor XIII-depleted human fibrinogen (American Diagnostica GmbH) was diluted in buffer (50 mM Tris, 100 mM NaCl and 5 mM EDTA) as indicated by the provider. The fibrinogen was mixed with human FXIII (American Diagnostica GmbH) and allowed to polymerize in the presence of human alpha-thrombin (American Diagnostica GmbH), CaCl_2_ (Sigma) and cell culture media FGM-2 BulletKit (Lonza). Finally, the hydrogels were hydrated and stored in an incubator for 24 h before initiating any experiment. The pH of the gels was 7.4, and the concentration of each constituent per final volume was 3.3 mg·mL^−1^ fibrinogen, 22 μg·mL^−1^ FXIII, 1 U·mL^−1^ thrombin and 5 mM CaCl_2_.

#### 2.1.2. Collagen Gels

The procedure for constructing collagen gels was adapted from a previous work by Shin *et al.* [46]. Collagen type I (BD Biosciences) was buffered to a final concentration of 2 mg·mL^−1^ with 10× DPBS (Gibco) supplemented with calcium and magnesium, cell culture media FGM-2 BulletKit (Lonza) and cell culture-grade water (Lonza). The pH of the dilution was adjusted to 7.4 with NaOH. Mixtures were allowed to polymerize inside humid chambers at 37 °C. Next, the gels were hydrated and stored in an incubator for 24 h before experimentation.

### 2.2. Scanning Electron Microscopy

Hydrogels were fixed with 2.5% glutaraldehyde (Sigma-Aldrich) followed by 1% electron microscopy grade osmium tetroxide (Ted Pella, Inc., Redding, CA, USA). The hydrogels were subsequently dehydrated in 30%, 50%, 70%, 80% and 95% ethanol solutions, respectively. Gels were freeze-fractured in liquid nitrogen before a final dehydration step in 100% ethanol. The gels were finally subjected to critical point drying using a Baltec CPD030. The samples were sputter-coated with gold-palladium for 4 min using an Emitech K550, resulting in a layer thickness of 15 nm. The samples were visualized by high-resolution imaging with a Merlin field emission scanning electron microscope (FESEM) from Zeiss with a beam voltage of 1 kV and a magnification of 80–120 kX.

### 2.3. Confocal Reflection Imaging

Confocal reflection was performed using a Leica SP2 equipped with a 63×/1.4 N.A. (numerical aperture) oil immersion lens. The samples were excited at 488 nm with an argon laser and detected at 479–498 nm.

### 2.4. Microstructural Analysis

To elucidate the microstructural features of the 3D networks, confocal reflection imaging (CRI) and SEM images were acquired as previously described. The void ratio, pore size and fiber radius were evaluated using the free software ImageJ [47]. For the fiber radius and pore size measurements, a straight line and measurement tools were employed. For void ratio analysis, confocal reflection images were binarized. The fiber-to-pore ratio was subsequently calculated from the areas of white (pores) and black (fibers) pixels within the binary images. Three independent sets were examined for each hydrogel, and the data are presented as the mean ± SEM.

### 2.5. Permeability Experiments

To measure Darcy’s permeability (K) of the hydrogels, a specific microfluidic-based experimental set-up was employed. This set-up reproduced the hydraulic environment of the *in vitro* physiological studies of wound healing. A microfluidic platform was used to assess the permeability values of both hydrogels.

**Figure 1:**
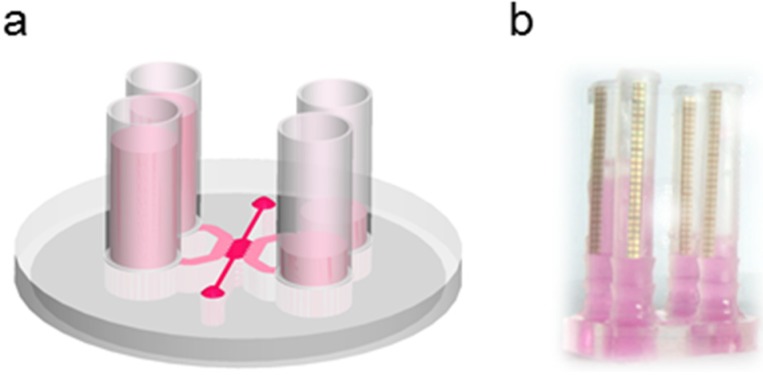
Microfluidic-based experimental set-up for permeability measurements. (**a**) The schematic shows the arrangement of the media columns with respect to the geometry. The design comprises a central gel cage (fuchsia) and two main media channels (pink), which are connected to the corresponding media columns. (**b**) A picture of an actual experiment demonstrates a pressure difference of approximately 100 Pa across the gel induced by the height difference between the media columns on both sides of the geometry.

The gels were allowed to polymerize within the microfluidic devices, which were fabricated as described by Shin *et al.* [46]. Medium reservoir tubes were inserted into the channel outlets (shown in Figure 1) as described by Sudo *et al.* [48]. The difference in height of the media columns on both sides of the gel caused a pressure gradient of 500 or 13 mm of H_2_O for fibrin and collagen, respectively.

From Darcy’s law, the relationship between the pressure difference and the permeability is as follows:

(1) $$∆\text{P}\left(\text{t}\right)=\;∆\text{P}\left(0\right)\cdot {\text{ e}}^{-\text{ct}}$$

where t is time, ∆P(t) is the pressure difference at each time point and ∆P(0) is the initial pressure difference. The constant c is related to the permeability K, as shown below [48]:

(2) $$\text{K}\left({\text{m}}^{2}\right)=\;\frac{\text{c}\cdot \text{μ}\cdot \text{L}\cdot A_{\text{r}}}{\text{ρ}\cdot \text{g}\cdot \text{A}}$$

where μ and ρ are the viscosity and density of the fluid, respectively, L is the length of the gel through which the pressure drop is established, $A_{r}$ is the area of the media reservoirs, g is the acceleration due to gravity and A is the cross-sectional flow area.

Therefore, Equation (1) states that, for a given initial pressure difference, the pressure difference will tend to equilibrium with an exponential decay. Based on this interpretation, by tracing the experimental pressure difference drop over time, the measured data points were fitted using Equation (1), and the value of c was obtained for each hydrogel. Finally, to characterize the interstitial resistance to flow, Equation (2) was solved for K, and Darcy’s permeability values were determined.

### 2.6. Rheology

For the rheological measurements, a Bohlin Gemini 200 HR Nano rheometer was used. The lower torque limit of the instrument was 3 nN·m in oscillation. All tests were performed using a cone-plate geometry with a diameter of 40 mm, a cone angle of 1° and a truncation height of 30 µm. The temperature was maintained at 37 °C ± 0.1 °C using a Peltier plate.

**Figure 2:**
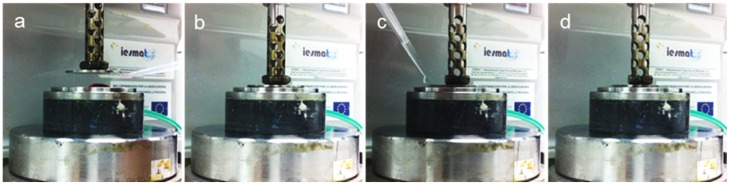
Image sequence of the gel filling process on the rheometer. Once the sample was mixed, it was pipetted *in situ* onto the rheometer plate (**a**); then, the gap was adjusted (**b**), and the sample was covered with oil (**c**) to prevent evaporation; the set-up was then ready to conduct the experiment (**d**).

The samples were pipetted onto the rheometer plate by filling its gap, as demonstrated in Figure 2. To prevent evaporation, the shear gap was covered with a 0.1 Pa·soil, used for calibration.

The curing reaction was traced by measuring the evolution of the shear modulus over time at a constant temperature of 37 °C, an oscillation frequency of 1 rad·s^−1^ and an applied strain amplitude of 0.005. The dependence of the sample moduli on the oscillatory strain amplitude was measured at a constant temperature of 37 °C for excitation frequencies of 0.1 Hz and 0.01 Hz. The strain amplitude was varied in a logarithmically-equidistant interval of 10 measurement points per decade from 0.001 to 1. For each point, data from 6 periods were accumulated.

## 3. Results and Discussion

### 3.1. Microstructural Study

**Figure 3:**
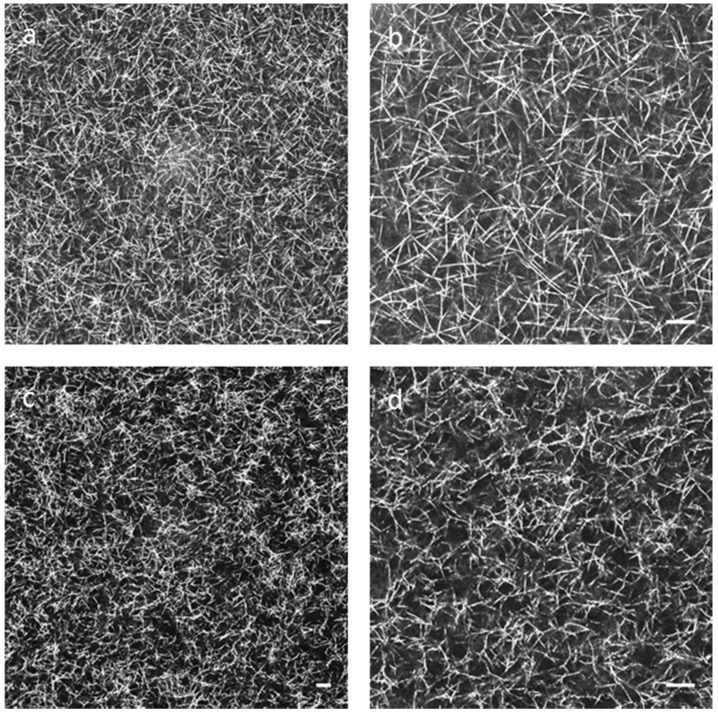
3D network of the hydrogels. The confocal reflection images show the arrangement of the fibrillar networks for (**a**,**b**) fibrin and (**c**,**d**) collagen gels. (**a**) and (**c**) show a general view; (**b**) and (**d**) are zoomed images of the right-bottom corner of the previous images, respectively. Fibers composing the collagen networks are twisted, whereas the fibers in the fibrin appear straighter. Scale bars correspond to 10 μm.

**Figure 4:**
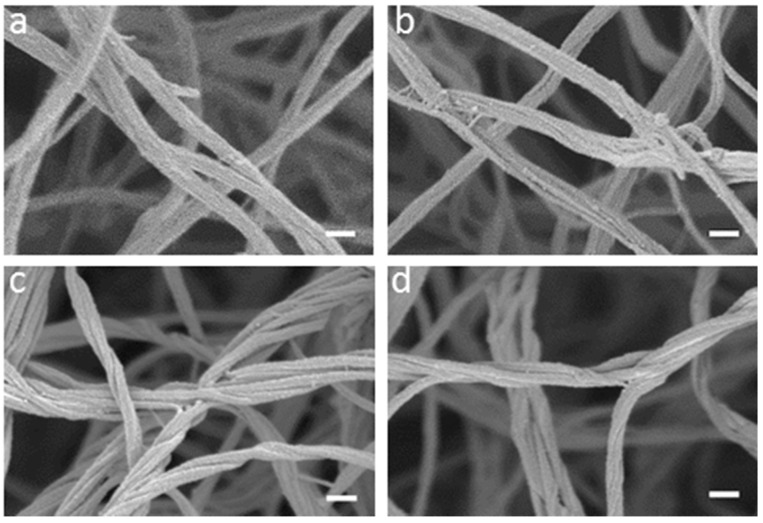
Fiber layout within hydrogels. SEM images of (**a**,**b**) fibrin and (**c**,**d**) collagen gels acquired at magnifications of 80–120 kX show the morphological features of the fibers. The collagen fibers exhibit characteristic bundling, whereas the fibrin fibers are formed more individually. The scale bars correspond to 200 nm.

Confocal reflection and SEM images were acquired to visualize both the collagen and fibrin hydrogels. As shown in Figure 3 and Figure 4, the lattices of fibrin were more entangled than those of collagen. The collagen networks exhibited twisted patterns formed by bundled fibers, consistent with that reported by Lai *et al.* [28,35]. The assembled fibrin fibers were straighter and appeared more individually, consistent with its role in physiological clot structures [49].

In addition to this qualitative assessment, microstructural features, such as the void ratio, pore size and fiber radius, were estimated from the images and are summarized in Table 1. As shown in the images, the fiber density of the fibrin networks was greater than that of the collagen matrices, leading to void ratios of approximately 71% and 80%, respectively. Accordingly, the pore size was 1.69 μm for fibrin and 2.84 μm for collagen. The fiber radii were approximately 79 and 66 nm for fibrin and collagen, respectively. For the collagen fibers, the variation of the measured data was quite high, probably due to the variability introduced by the characteristic bundling.

**Table 1:** Microstructural characteristics of the hydrogels ^†^.

	Collagen	Fibrin
Void ratio (%)	80.15 ± 1.82	71.46 ± 1.00
Pore size (μm)	2.84 ± 0.94	1.69 ± 0.33
Fiber radius (nm)	79.51 ± 33.16	66.53 ± 13.57

^†^ Data are the mean ± SEM.

### 3.2. Permeability Quantification

Previous work has primarily focused on analyses of microstructure and bulk stiffness due to the key roles of these properties in basic cellular events in 2D [50]. However, wound healing primarily occurs under 3D conditions, and there is accumulating evidence that the confinement of cells has a crucial role on their behavior [21,22,51,52]. A bulk quantity that relates to the confinement feature of hydrogels is their hydraulic resistance. The hydraulic resistance of hydrogel matrices not only controls the transport of nutrients and the shear stress exerted on cells, but also regulates the directional migration of the cells [42,53]. Therefore, in this characterization, we include the assessment of the hydraulic resistance to fluid flow of the scaffolds, because it is key to the rational design and interpretation of physiologically-relevant microsystems.

**Figure 5:**
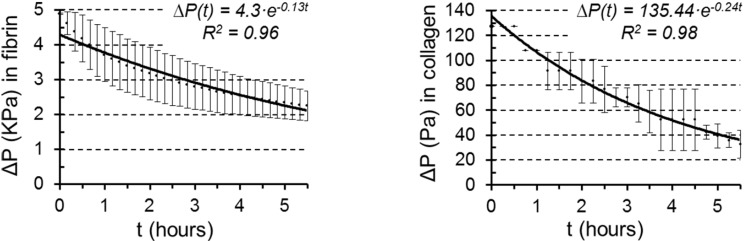
Pressure difference drop over time in hydrogels. The experimental data points of the pressure difference *vs.* time were plotted and fitted to an exponential function for fibrin (**left**) and collagen (**right**) gels. The resulting expressions and *R^2^* values are also indicated. The exponent coefficient determines the value of the permeability (K).

**Figure 6:**
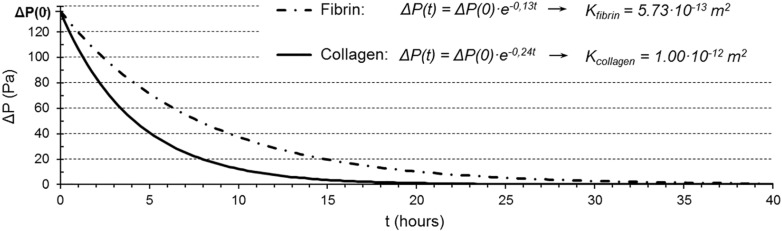
Comparison of the pressure difference drop for both hydrogels. The curves show the pressure difference drop over time for a given initial pressure difference for the collagen and fibrin hydrogels. Compared with collagen, the lower permeability (*K*) of the fibrin results in a slower function decay.

To quantify the hydrogel permeability, we generated an initial pressure difference across both hydrogels. As shown in Figure 5, the decrease in pressure difference over time was then monitored. The obtained data points were then fitted to an exponential function formatted as in Equation (1), and *R^2^* values of 0.96 and 0.98 for fibrin and collagen, respectively, were obtained. The exponent coefficient values (c) from the fitting were 0.13 h^−1^ or 4.00·10^−05^ s^−1^ for fibrin and 0.24 h^−1^ or 7.00·10^−05^ s^−1^ for collagen. Likewise, by solving Equation (2) for K, we calculated Darcy’s permeability for fibrin and collagen: 5.73·10^−13^ and 1.00·10^−12^ m^2^, respectively. The values of c and K are provided in Table 2. These parameters establish the resistance that a particular porous matrix exerts on the convective fluid flow, *i.e.*, the velocity with which a pressure difference will tend toward equilibrium. Figure 6 illustrates, for a given initial pressure difference, the pressure difference decay for both hydrogels. Pressure equilibrium is achieved more rapidly in the collagen gels than the fibrin gels, indicating less resistance to flow and consistent with the greater void ratio and pore size of the collagen gel observed in the image analysis and other previous measurements [41,42].

**Table 2:** Resistance to fluid flow.

	Collagen	Fibrin
Exponent coefficient, c (s^−1^)	7.00·10^−05^	4.00·10^−05^
Darcy’s permeability, K (m^2^)	1.00·10^−12^	5.73·10^−13^

### 3.3. Mechanical Response

**Figure 7:**
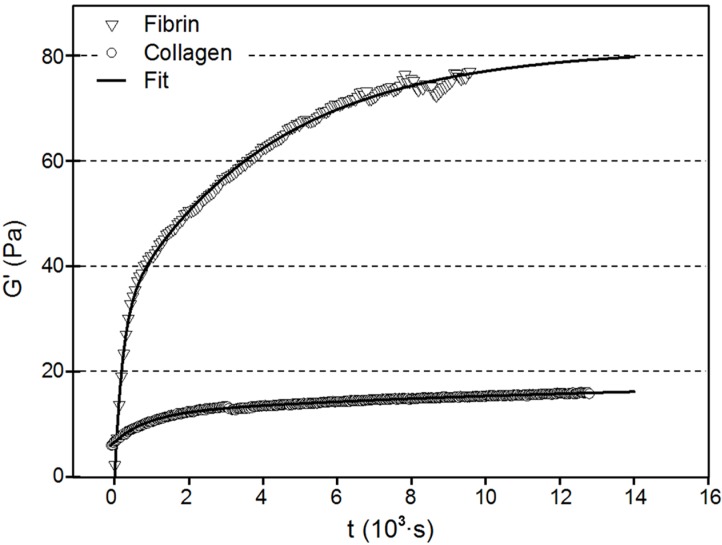
Time evolution of the shear modulus (*G’*) of the hydrogels. The temperature was maintained at 37 °C; the excitation frequency was 1 rad·s^−1^; and the strain amplitude was 0.005. The lines are fits to the biexponential Equation (3). Note that the polymerization of the gels was not yet complete, *cf.* Figure 8.

The polymerization was traced for 3 h, beginning immediately after the gel solution was pipetted and set within the rheometer plate. Figure 7 shows the evolution of the shear modulus (*G’*) over time at a constant temperature of 37 °C. The biexponential function given in Equation (3) fits the measured data points well, thus suggesting two processes with distinct reaction rates (a fast and a slow rate), *t*_1_ and *t*_2_, resulting in an increase in the value of the modulus:

(3) $$G^{\prime }\left(t\right)={G^{\prime }}_{1}\left(1-e^{-\left(\frac{t-x_{c}}{t_{1}}\right)}\right)+{G^{\prime }}_{2}\left(1-e^{-\left(\frac{t-x_{c}}{t_{2}}\right)}\right)$$

where *G’*_1_ and *G’*_2_, are the associated parameters that characterize the modulus contribution of each process and *x_c_* is the parameter that adjusts the time scale to the beginning of the reactions.

The fitted parameter values are shown in Table 3. The fast and slow processes may be associated with the fibrillogenesis of the filaments (fast) and the growth and crosslinking of these filaments (slow) to form a fiber network. Clearly, fibrin cured faster than collagen under the conditions studied and achieved significantly greater modulus values. Furthermore, the fast and the slow processes are more easily distinguishable in fibrin compared with collagen. Fibrin gels are cross-linked and stabilized by FXIII; accordingly, the complete cross-linking of a blood clot during coagulation takes longer than the formation of its fibrillar backbone [29], which could explain the differences in the polymerization kinetics of the hydrogels (see Figure 7). This fact could also explain why confocal scanning micrographs do not show much difference with or without ligation [38].

**Table 3:** Parameters used to fit Equation (3) to the measured data in Figure 7.

	Fibrin		Collagen	
	*Value*	*Error*	*Value*	*Error*
*G*’_1_	31.12	0.48	11.70	0.10
*t*_1_	211.87	11.09	896.75	22.16
*G*’_2_	50.78	0.35	7.11	0.30
*t*_2_	4,045.83	113.48	13,919.40	1,431.71
*x_c_*	117.52	4.63	−591.82	15.62

**Figure 8:**
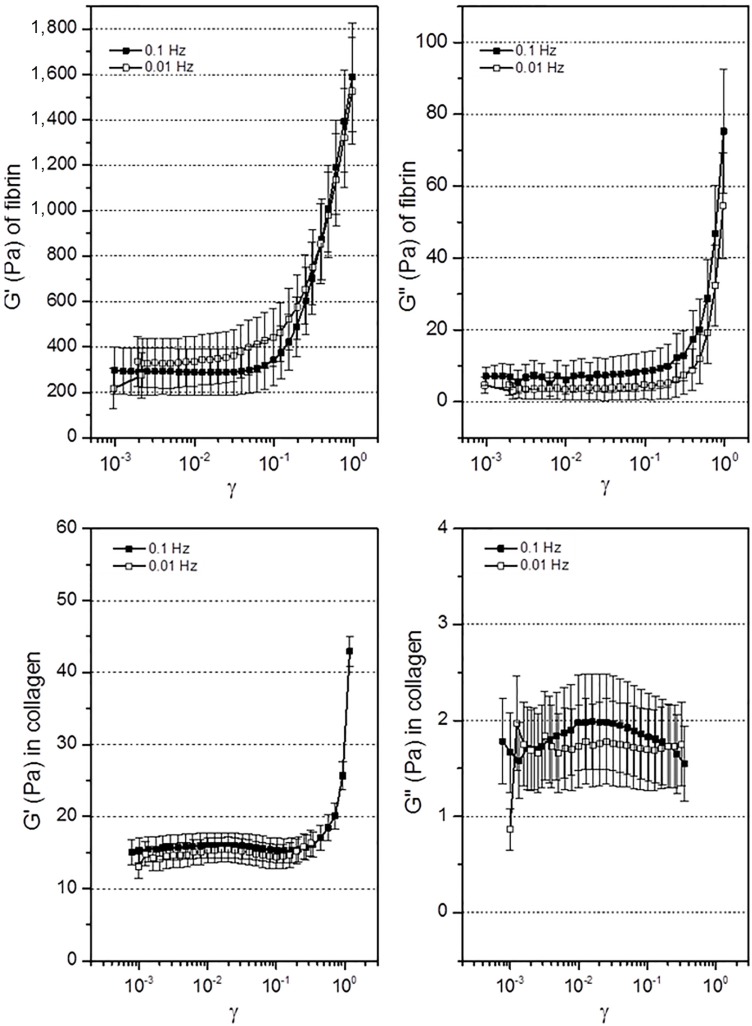
Strain sweeps of hydrogels. Elastic (*G’*) and viscous (*G’’*) shear moduli are shown as a function of the strain amplitude (*γ*) in parts per unit at frequencies of 0.1 Hz and 0.01 Hz. The temperature during the experiments was maintained at 37 °C. Strain hardening of fibrin and collagen occurred at 10% and 50% strain, respectively. In the elastic range, the elastic shear modulus was 300 and 15 Pa, respectively.

After tracing the curing, the samples remained at rest *in situ* for 24 h before initiating any subsequent rheological experiments. Strain sweep assays were performed for excitation frequencies of 0.1 and 0.01 Hz. Figure 8 outlines the dependence of the measured elastic (*G’*) and viscous (*G’’*) shear moduli on the applied strain amplitude for the fibrin and collagen gels. For the measured excitation frequencies, the registered moduli were similar within the experimental error. The mechanical response of the hydrogels tested by means of oscillatory strain amplitude sweeps revealed the shear modulus in the linear viscoelastic regime and the onset of strain hardening at greater strains (non-linear regime) of the individual hydrogels.

In the linear viscoelastic regime, for the fibrin networks, we measured an elastic shear modulus of 300 Pa, which matches other published values [27,29,33,45]. For the collagen gels, a value of 15 Pa was determined. These data agree with previous measurements obtained using analogous gel preparations [30,54]. Comparing fibrin to collagen, we attributed the dramatic increase in stiffness to FXIII, consistent with its role in acute clots to prevent bleeding problems [49], because the protein concentration difference between the hydrogels and the difference in the microstructural parameters appeared too small to explain the difference in modulus alone.

Both materials were characterized by substantial strain hardening, which occurred within the strain range of 10%–100% and 50%–100% for fibrin and collagen, respectively. On a physical level, the strain hardening of biological hydrogels can be interpreted in analogy to the polymer network theory of semiflexible chains. Semiflexible chains are characterized by similar magnitudes of persistence length and contour length. In networks, the relevant contour length is the distance between network junctions. Such semiflexible chains do not form loops and knots, yet are sufficiently flexible to have significant thermal bending fluctuations [30]. Therefore, in a simplified picture, the onset of strain hardening relates to the straightening of these semiflexible filaments in the network upon straining. An earlier onset of strain hardening suggests a lower degree of freedom and lower thermal fluctuations as a result of a greater ratio of the persistence to the contour length or, in other words, a straighter filament.

It is tempting to associate the straighter appearance of the fibrin filaments relative to the collagen filaments, as observed in the confocal reflection images (*cf.*, Figure 3), with the somewhat earlier onset of strain hardening in the fibrin and, consequently, to the greater ratio of the persistence to the contour length in the fibrin hydrogel.

In fact, strain hardening is generic to any network composed of semiflexible filamentous proteins [29,30,38,55]. Many soft tissues, such as blood clots, stiffen as they are strained to prevent large deformations that could threaten tissue integrity [30,56,57]. Therefore, strain hardening performs an essential physiological function. Moreover, during wound healing *in vivo*, the ECM is exposed to repeated strain due to cellular contractile machinery, cellular motility, blood flow or interstitial flow [29]. Likewise, at a cellular level, cells embedded in 3D exert forces that cause deformations of 10%–50% in the cell surroundings (20 microns far from the cell margin) and 10% away from the cell [58]. Therefore, we assume that, during wound healing, cells settle their contractile activity within the linear range. In addition, the characterization of the strain hardening and its onset is, in our opinion, an important parameter to control the creation of biomimetic hydrogels for wound healing applications.

## 4. Conclusions

We have presented a consistent characterization of two widely-used hydrogel compositions based on fibrin and collagen I as biomimetic environments for *in vitro* wound healing studies. The microstructural parameters obtained from microscopic techniques that probed the local and 2D environments correlate with the permeabilities and mechanical values measured using experimental techniques capable of measuring bulk properties in 3D. The studied fibrin hydrogel is characterized by a lower void ratio, lower permeability and a significantly greater shear modulus compared with the collagen hydrogel.

Distinct biomechanical properties differentially regulate migration in 2D and 3D [59]. Although 2D system mechanisms are generally established, the effects of different biomechanical properties of hydrogel materials, such as matrix stiffness [60], microarchitecture [61] or confinement [22], on 3D migratory patterns remain to be elucidated. In this sense, the presented functional characterization of the two widely-used fibrin and collagen hydrogels provides complementary and coherent experimental parameters for the biomechanical properties of the studied hydrogels as a basis for an interpretation of cellular studies in 3D for wound healing experiments. Therefore, the data presented here will open new possibilities for future models, both *in silico* and *in vitro*, of the main mechanisms that regulate wound healing. Furthermore, microfluidic systems, which are biomimetic 3D models in which hydrogels are embedded, will provide multiple possibilities for improving the recreation of the wound healing microenvironment and enable the incorporation of cell cultures, as well as co-cultures of different cell types.

Finally, our experimental approach provides a method for the measurement of the most relevant biomechanical properties of hydrogels, enabling a systematic study of the influence of the numerous combinations of compositions and preparation conditions on these scaffolds and, consequently, cell behavior.
